# Supplementary material for: Hydrogen bonds to Au atoms in coordinated gold clusters
Source: Nat Commun. 2017 Sep 18;8:576. doi: 10.1038/s41467-017-00720-3 (PMC5603579; doi:10.1038/s41467-017-00720-3)
Supplement: Supplementary file 1 — Supplementary Information [file 41467_2017_720_MOESM1_ESM.pdf]

Supplementary Methods

Materials. Tetrachloroauric(III) acid tetrahydrate (>47.5% for gold) was obtained from Tanaka Kikinzoku kogyo. Tetrahydrofuran (super-dry, 99.5%), diethyl ether (super-dry, 99.5%), *n*-butyllithium in hexane (1.6 mol/L), methanol (99.5%), ethanol (99.5%), dichloromethane (99%) and diethyl ether (99%) were purchased from Kanto chemicals. 1,3-Dibromobenzene (97%) and potassium hexafluorophosphate (95%) were obtained from TCI. 1,3-Bis(diphenylphosphino)propane (TMDP, 97%) and chlorodiphenylphosphine (99%) were purchased from Aldrich. All the chemicals and reagents were used as received.

Supplementary Table 1. Crystal data, data collection and structure refinement for **1**·$(PF_6)_2$·$4CH_2Cl_2$

Empirical formula	C124 H104 Au6 Cl8 F12 P10
Formula weight	3597.17
Temperature	90 K
Wavelength	0.71073 Å
Crystal system	monoclinic
Space group	P 1 21/c 1
Unit cell dimensions	a = 27.6425(17) Å α = 90°
	b = 26.6486(16) Å β = 101.2133(8)°
	c = 16.9880(11) Å γ = 90°
Volume	12275.0(13) $Å^3$
Z	4
Density (calculated)	1.946 g/m^3
Absorption coefficient	7.516 mm^{-1}
F(000)	6864
Crystal size	0.42 x 0.30 x 0.18 mm^3
Theta range for data collection	1.07 to 26.73°
Index ranges	$-32 \le h \le 34$, $-33 \le k \le 33$, $-14 \le l \le 21$
Reflections collected	67149
Independent reflections	26013 [R(int) = 0.0250]
Reflections with I > 2□(I)	20774
Completeness to theta = 26.73°	99.8 %
Absorption correction	Multi scan
Max. and min. transmission	0.3400 and 0.1900
Refinement method	Full-matrix least-squares on F^2
Data / restraints / parameters	20774 / 1492 / 1441
Goodness-of-fit on F^2	1.080
Final R indices [I > 2□(I)]	R_1 = 0.0569, wR_2 = 0.1377
R indices (all data)	R_1 = 0.0756, wR_2 = 0.1487
Largest diff. peak and hole	2.817 and -2.607 $eÅ^{-3}$

Supplementary Table 2. Crystal data, data collection and structure refinement for **2**·$(NO_3)_2$·Et_2O·$4H_2O$.

Empirical formula	C116 H130 Au6 N2 O11 P8	
Formula weight	3157.78	
Temperature	90 K	
Wavelength	0.71073 Å	
Crystal system	Orthorhombic	
Space group	Pbcn	
Unit cell dimensions	a = 25.6660(12) Å	α = 90°
	b = 34.0155(16) Å	β = 90°
	c = 26.7868(13) Å	γ = 90°
Volume	23386.0(19) $Å^3$	
Z	8	
Density (calculated)	1.794 g/m^3	
Absorption coefficient	7.666 mm^{-1}	
F(000)	12176	
Crystal size	0.36 x 0.09 x 0.05 mm^3	
Theta range for data collection	0.99 to 26.02°	
Index ranges	$-25 \leq h \leq 31$, $-40 \leq k \leq 41$, $-33 \leq l \leq 29$	
Reflections collected	118118	
Independent reflections	23036 [R(int) = 0.0250]	
Reflections with I > 2□(I)	15284	
Completeness to theta = 26.02°	100 %	
Absorption correction	empirical	
Max. and min. transmission	0.1689 and 0.7005	
Refinement method	Full-matrix least-squares on F^2	
Data / restraints / parameters	23036 / 666 / 1200	
Goodness-of-fit on F^2	1.091	
Final R indices [I > 2□(I)]	R_1 = 0.0466, wR_2 = 0.1188	
R indices (all data)	R_1 = 0.0952, wR_2 = 0.1506	
Largest diff. peak and hole	2.958 and -2.397 e$Å^{-3}$	

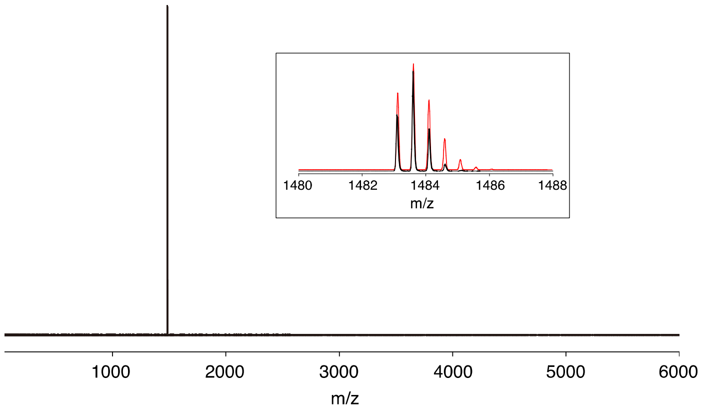

Supplementary Figure 1. ESI-MS spectrum of **1**·$(PF_6)_2$ (Inset) Expanded view (black) superimposed with a simulated spectrum (red).

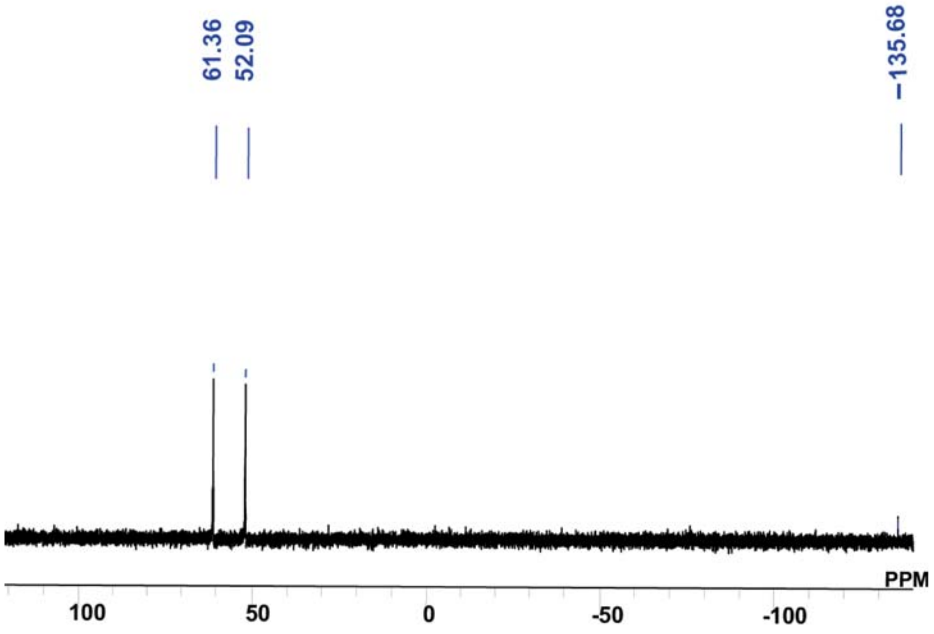

Supplementary Figure 2. ^{31}P NMR spectrum of **1**·$(PF_6)_2$ in CD_2Cl_2 at 25 °C.

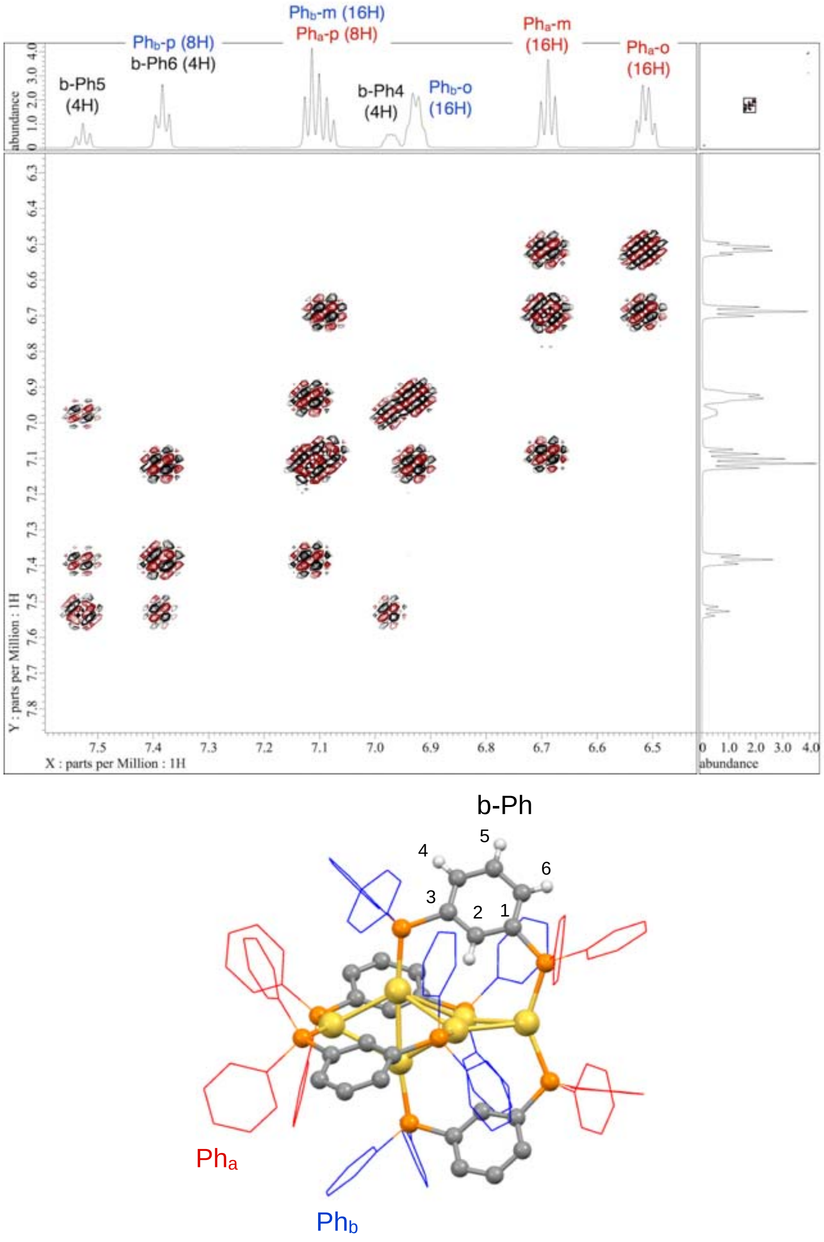

Supplementary Figure 3. Aromatic region of ^{1}H-^{1}H DQF-COSY (600 MHz) spectrum of **1**$\cdot(PF_6)_2$ in CD_2Cl_2 at room temperature (~ 24 °C). Structure of **1** with atom/group labeling is also shown for the peak assignments. The assignments for Ph_a / Ph_b and b-Ph4 / b-Ph6 in Supplementary Figures 3 – 5 are uncertain.

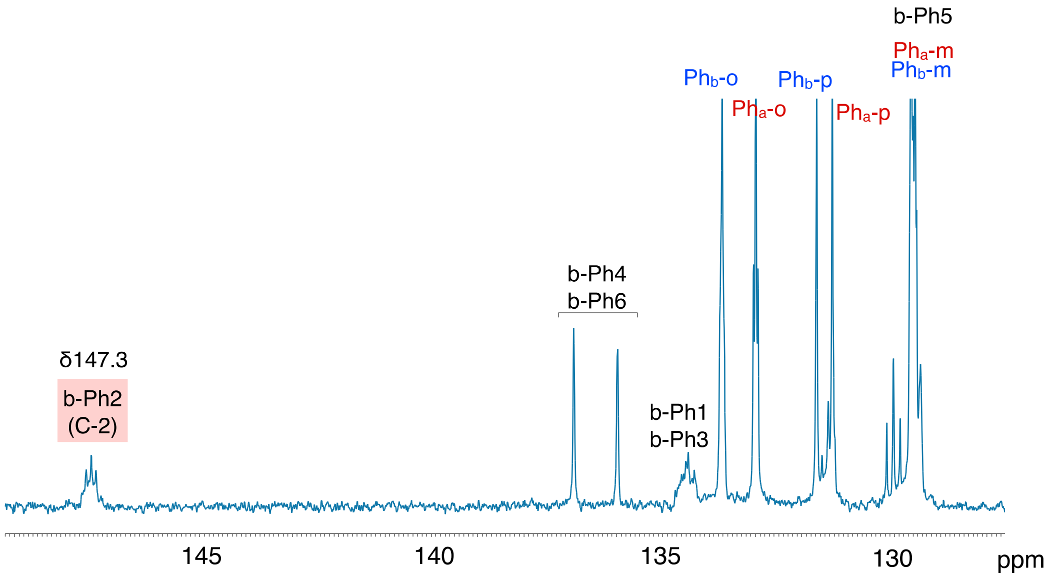

Supplementary Figure 4. Aromatic region of ^{13}C NMR (150 MHz) spectrum of **1**·$(PF_6)_2$ in CD_2Cl_2 at room temperature (~ 24 °C). For the peak assignments, see the structure of Supplementary Figure 3.

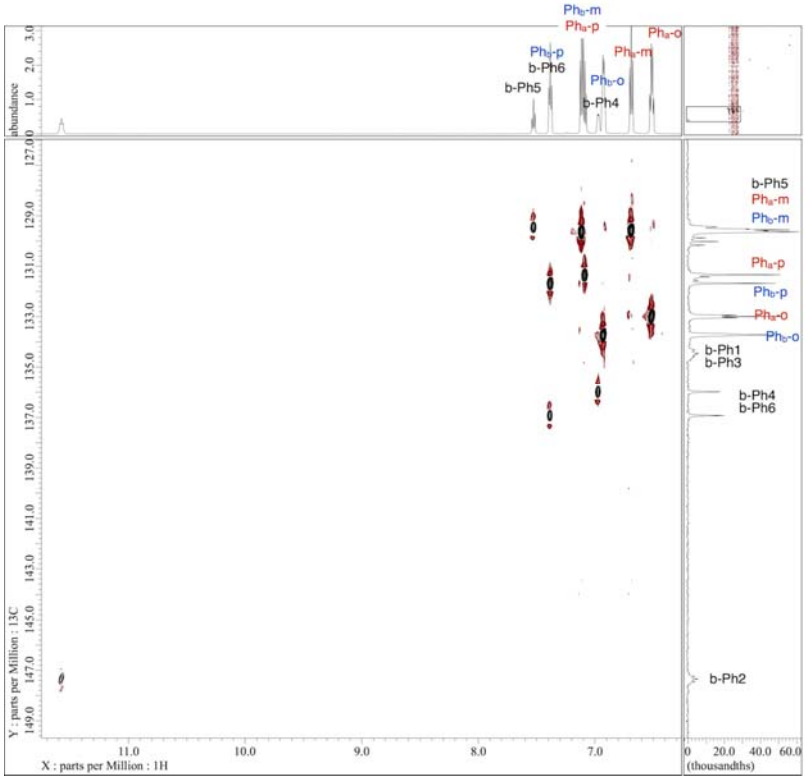

Supplementary Figure 5. HSQC (600 MHz) spectrum (aromatic region) of **1**·$(PF_6)_2$. in CD_2Cl_2 at room temperature (~ 24 °C). For the peak assignments, see the structure of Supplementary Figure 3.

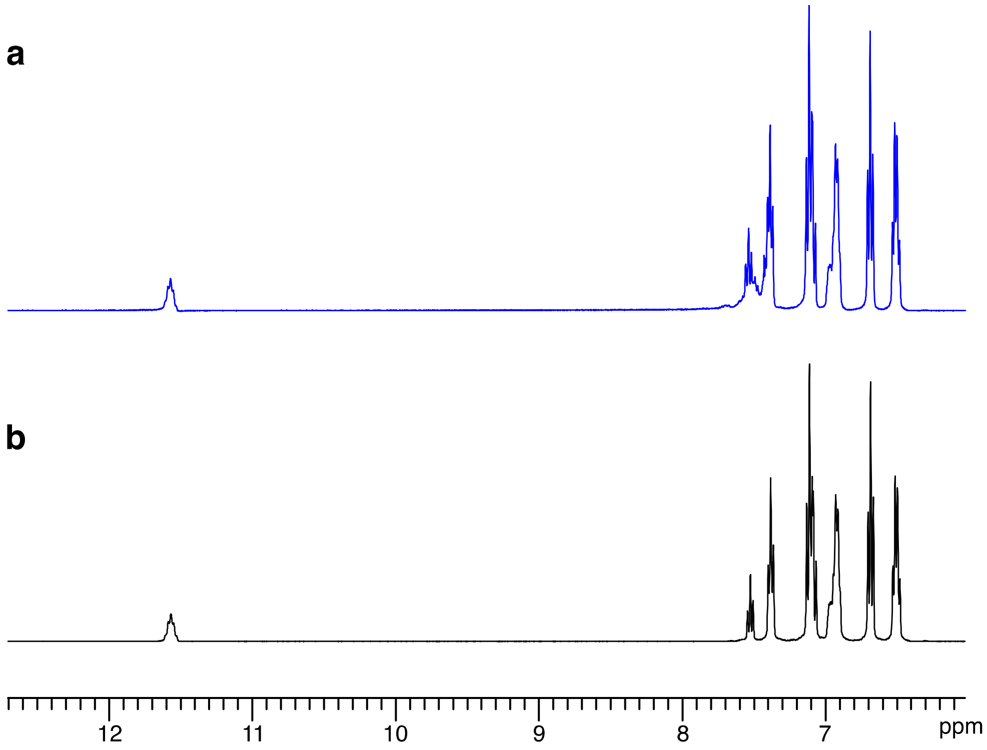

Supplementary Figure 6. ^{1}H-NMR (400 MHz) spectra of **a**) **1**·$(NO_3)_2$ and **b**) **1**·$(PF_6)_2$ in CD_2Cl_2 at room temperature (~ 24 °C).

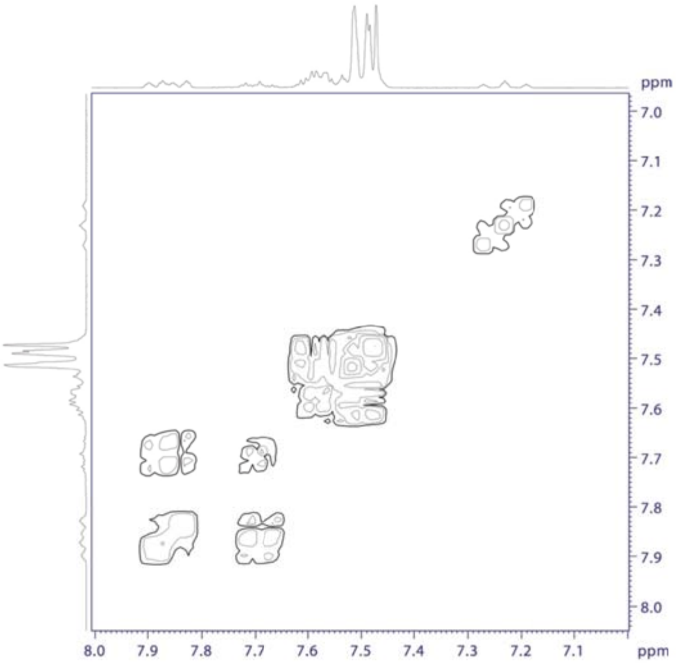

Supplementary Figure 7. ^{1}H-^{1}H DQF-COSY (400 MHz) spectrum (aromatic region) of Au_2(*m*PhDP)$_2Cl_2$ (**3**) in CD_2Cl_2 at room temperature (~ 24 °C).

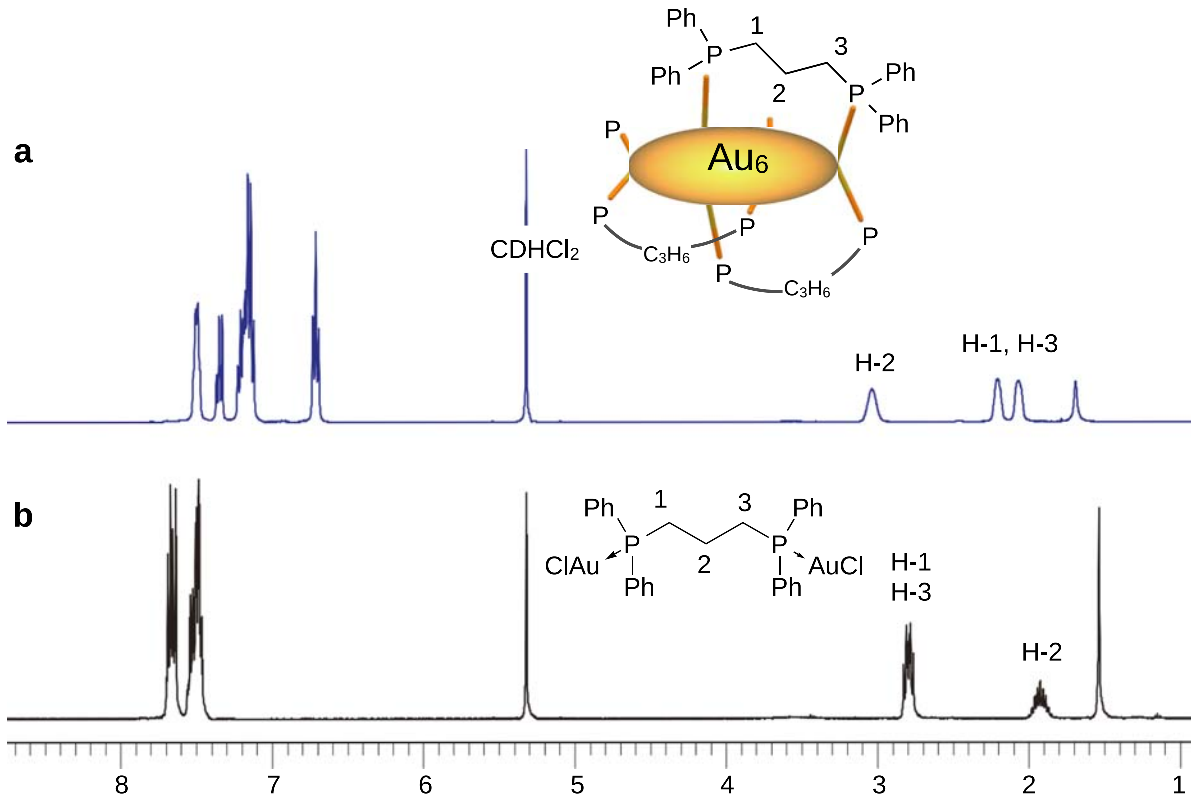

Supplementary Figure 8. ^{1}H NMR spectra (400 MHz) of **a**) **2**·$(NO_3)_2$ and **b**) **4** in CD_2Cl_2 at ~ 24 °C.
